# Hospital Treatment for Insured Persons, and Mr. Lloyd George's Bill

**Published:** 1911-11-11

**Authors:** 


### The Hospital

The Workers' Newspaper of Administrative Medicine and Institutional Life, Administration, National Insurance and Health.

No. 1322, Vol. LI. SATURDAY, NOVEMBER 11TH, 1911. PRICE ONE PENNY.

### HOSPITAL TREATMENT FOR INSURED PERSONS, AND MR. LLOYD GEORGE'S BILL.

We are glad to be in a position to report that the voluntary hospitals and the representatives of the medical profession are at one in maintaining that hospital treatment for insured persons must be included in the National Insurance Bill before it becomes an Act of Parliament. The claim for National Insurance in the United Kingdom, as in Germany, rests upon the guarantee of its promoters, as expressed in public speeches in and out of Parliament, that *its purpose is to ensure a certain and sufficient relief in case of illness for the insured.* In introducing the Bill, Mr. Lloyd George claimed as one of its great merits that the benefits offered to the insured included free medical relief with no taint of charity. These two points must be kept continuously in mind, for the Bill, if it were to pass without the inclusion of hospital treatment for the insured, must prove in practice a tragedy by inflicting grievous injury on the insured when seriously ill.

That the position here taken up is sober, purposeful sense, without exaggeration, must become perfectly clear when the actual facts are examined. The Insurance Bill will create 15,000,000 insured persons who will represent 36 per cent. of the population of the United Kingdom. The highest authorities, backed by experience, prove that the hospital provision required to meet the actual in-patient needs of a given population is two hospital beds for every 1,000 people. It follows that the insured under Mr. Lloyd George's Bill will require each year for their continuous use 30,000 hospital beds. That is to say, that, to fulfil the claim of its promoters, this Bill must put the Insurance Commissioners in a position through the Health Committees to command the use of 30,000 hospital beds each year for the exclusive use of the 15,000,000 insured persons created by it. Otherwise the claim of its promoters, that its avowed purpose is to ensure to every one of these participants a certain and sufficient relief in case of illness, will be demonstrably proved to be without foundation. Further and worse, in such circumstances the lot of all these 15,000,000 insured persons under the Bill when they are grievously ill and require hospital care must be infinitely worse than it is at present without any scheme of national insurance whatever.

The more this question of hospital treatment under this Bill is examined, the more serious it becomes. Take London as an example of what may happen should the Bill pass without the inclusion of hospital treatment. Approximately 2,500,000 Londoners will be insured, and they will require for their sole use and treatment 5,000 hospital beds. The voluntary and endowed hospitals of London provide approximately 10,500 beds, of which nearly 10,000 are continuously occupied. The voluntary hospitals by their constitution cannot without grave abuse grant free in-patient treatment to an insured person, because every such person in virtue of his own payments, and the payments made on his behalf by employers and the State, *is guaranteed by the Government a certain and sufficient relief in case of illness.*

We have shown in previous articles how gravely this guaranteed provision for 15,000,000 members of the working classes may affect the revenues, from voluntary sources, of our voluntary hospitals. Taking the country altogether it is clear that on an average at least half the whole income at present devoted to the maintenance of the voluntary hospitals may be imperilled or lost if infinite care is not taken to prevent it at this stage before the passing of the National Insurance Bill. These figures have been given in detail and in bulk. They are demonstrably provable from the audited accounts available, and no auditor or arbitrator on the evidence, being a just man, could put the damage at much less than half the total revenue of the voluntary hospitals. That is to say, that the voluntary hospitals on the passing of this Bill may speedily have only sufficient revenue to maintain half the number of beds which they at present provide for the solace of the general poor who are sick and suffering. If that be so, taking the case of the Metropolitan hospitals, assuming that Parliament could be got to agree to leave the insured without any hospital provision whatever, the voluntary hospitals could not give that provision in the form of free relief, for if they did there might not be a single bed available for the reception of the poor people who actually have the first claim to every such bed under the constitution of each voluntary hospital. This constitution is based upon the fact that such patients are themselves unable to defray the cost of sickness and accident in their own case and of members of their family. In a nutshell then the Bill as at present drawn may, and probably will, have the effect of recruiting 2,500,000 of insured persons in London who will require for their adequate treatment the continuous use of 5,000 hospital beds, and at the same time may diminish the present number of available beds in the London hospitals by 5,000. The same results, *ceteris paribus*, can be worked out in regard to other urban populations throughout the United Kingdom.

It appears certain therefore that Mr. Lloyd George and his advisers must have overlooked these momentous facts. When they are realised we have little doubt that the prayer of the managers of the voluntary hospitals and of the medical profession will be granted, and that hospital treatment will be included in the National Insurance Bill, preferably on a *pro rata* basis, by arrangements through the Insurance Commissioners with individual hospitals up and down the country.

